# Role of the F-BAR Family Member PSTPIP2 in Autoinflammatory Diseases

**DOI:** 10.3389/fimmu.2021.585412

**Published:** 2021-06-28

**Authors:** Jie-Jie Xu, Hai-Di Li, Xiao-Sa Du, Juan-Juan Li, Xiao-Ming Meng, Cheng Huang, Jun Li

**Affiliations:** Inflammation and Immune Mediated Diseases Laboratory of Anhui Province, Anhui Institute of Innovative Drugs, School of Pharmacy, Anhui Medical University, Hefei, China

**Keywords:** proline-serine-threonine-phosphatase-interacting protein 2, autoinflammatory diseases, macrophages, osteoclast, PEST-PTPs

## Abstract

Proline-serine-threonine-phosphatase-interacting protein 2 (PSTPIP2) belongs to the Fes/CIP4 homology-Bin/Amphiphysin/Rvs (F-BAR) domain family. It exhibits lipid-binding, membrane deformation, and F-actin binding activity, suggesting broader roles at the membrane–cytoskeleton interface. PSTPIP2 is known to participate in macrophage activation, neutrophil migration, cytokine production, and osteoclast differentiation. In recent years, it has been observed to play important roles in innate immune diseases and autoinflammatory diseases (AIDs). Current research indicates that the protein tyrosine phosphatase PTP-PEST, Src homology domain-containing inositol 5’-phosphatase 1 (SHIP1), and C‐terminal Src kinase (CSK) can bind to PSTPIP2 and inhibit the development of AIDs. However, the mechanisms underlying the function of PSTPIP2 have not been fully elucidated. This article reviews the research progress and mechanisms of PSTPIP2 in AIDs. PSTPIP2 also provides a new therapeutic target for the treatment of AIDs.

## Introduction

The concept of “autoinflammatory diseases (AIDs)” was first proposed in 1999 (1). AIDs arise from chronic activation of B and T cells of the innate immune system (2, 3), which begin to attack the body’s own tissues, causing loss of function in the composite organs and resulting in diseases. And monocytes, macrophages and neutrophils are the main cell types of the innate immune system (4).

AIDs are characterized by excessive apoptosis, hyperactive inflammatory cytokine production, and an overreaction to chemotactic stimuli (5), resulting in chronic, systemic inflammation. The field of AIDs has become a cornerstone of modern medicine, yet remains a growing area of research. Interestingly, many AIDs result from single gene mutations. For example, Familial Mediterranean fever (FMF) is an autosomal recessive self-inflammatory disorder caused by a mutation in the gene encoding MEFV (4). *LPIN2* mutation causes Majeed syndrome, characterized by chronic recurrent multifocal osteomyelitis (CRMO) (6, 7). More than 60 genes linked to AIDs have been discovered that affect distinct parts of the innate immune system, and it has been found that there are multiple genetic and environmental influences on the development of AIDs, which modulates the phenotype (8), such as behcet disease (BD) (9) and adult-onset still’s disease (AOSD) (10).

Proline-serine-threonine-phosphatase-interacting protein 2 (PSTPIP2) belongs to the F-BAR family of proteins and coordinates the function of actin in the cytoskeleton, including membrane degeneration, filopodia formation, movement, and surface adhesion, and is mainly expressed in macrophages (11). Currently, PSTPIP2 has been shown to play important roles in autoinflammatory diseases, including chronic recurrent multifocal osteomyelitis (CRMO) (12, 13) and sepsis (14, 15). PSTPIP2 absence causes autoinflammatory diseases, including extramedullary hematopoiesis, as verified by expansion of macrophage progenitors. PSTPIP2 knockdown mice also show skin and bone injury and mimic human multiple osteomyelitis (16, 17). Recent studies have shown that PSTPIP2 also plays important roles in liver fibrosis (18) and cisplatin-induced acute kidney injury (19), and is a predictive target for many other diseases, such as tuberculosis (20) and asthma (21). PSTPIP2 also inhibits osteoclast differentiation and reduces osteoclast ability to inhibit megakaryocyte differentiation (22, 23). This article will review the study of PSTPIP2 with respect to its roles in inflammatory diseases.

## Structure and Biological Function of PSTPIP2

The F-BAR protein was originally identified as CDC42-interacting protein 4 (CIP4). The N-terminal region of CIP4 is highly conserved in other proteins, including tyrosine kinase FES and FES related (FER), which is termed FES/CIP4 homology (FCH) domain, also called an EFC (an extended FCH domain) that interact with phospholipids, particularly PtdIns (4,5) P2, and induce membrane tubulation (24, 25).

F-BAR proteins have been considered as important new coordinating proteins that not only regulate endocytosis and phagocytosis, but also the filopodium, lamellipodium, cytokinesis, adhesion, and podosome formation (25). Existing data indicate that F-BAR proteins are membrane-related proteins that regulate membrane curvature by binding to cell membrane phospholipids, indicating that they have a wider range of effects at the membrane-cytoskeleton interface (24, 26, 27), and this function is in line with their participation in exocytosis (28, 29), endocytosis (26, 30–32) and endosomal recycling (33). The F-BAR family proteins can also participate in macrophage activation, neutrophil migration, cytokine production, and osteoclast differentiation (17, 23, 27, 34).

The F-BAR family proteins have been reported to regulate many cellular functions *via* F-actin assembly (11). It has been shown that the EFC/F-BAR domain dimers are joined end-to-end to form filaments for membrane-varus and endocytosis (35). The F-BAR domain can connect the cytoskeleton and cell membrane by binding to the negatively charged membrane phospholipids in the lipid membrane (36). Importantly, this oligomerization enables F-BAR family proteins to interact with different proteins simultaneously to form a larger branched protein complex.

The mutations in PSTP1P1 and PSTPIP2 have been found in association with AIDs, respectively in human and mice (37, 38). Previous reports have demonstrated that mutations in PSTPIP1 result in human pyogenic sterile arthritis, pyoderma gangrenosum, pyogenic arthritis, pyoderma gangrenosum, and acne syndrome, a dominant autosomal autoinflammatory diseases (37). PSTPIP1-associated myeloid-related proteinemia inflammatory syndrome (PAMI) has been described as a unique clinical phenotype of PSTPIP1-associated inflammatory disease (PADS) (39).

The cmo (L98P) and Lupo (I282N) mutations of PSTPIP2 in mice lead to macrophage-mediated autoinflammatory diseases, including skin necrosis, inflammation of the claw and ear, and inflammatory bone resorption (17, 38, 40). Mutations to PSTPIP2 in mice, induced by N-ethyl-N-nitrosourea, lead to phenotypes parallel to cmo and Lupo (41). Mice with cmo and Lupo in the early stages of disease exhibited markers of macrophage activation by increasing levels of inflammatory cytokines MIP-1 and IL-6 (16, 17).

**Figure 1:**
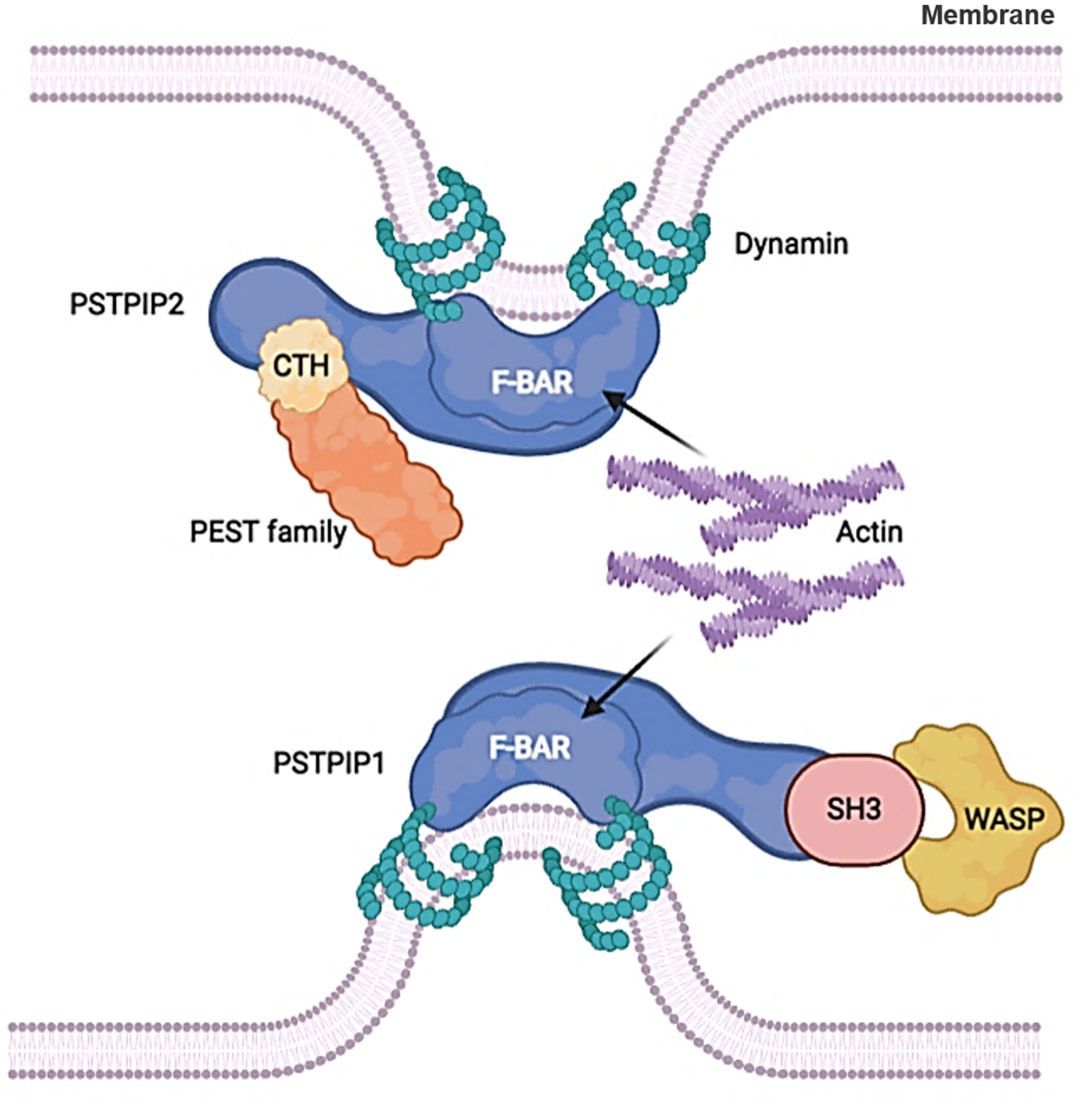
F-BAR proteins regulate membrane curvature by binding to cell membrane phospholipids. PSTPIP2 lacks the SH3 domain that is indispensable for interaction with WASP compared with PSTPIP1. Instead, it interacts with the C-terminal homology domain of the PEST family phosphatases.

Many F-BAR family proteins include the C-terminal Src homology 3 (SH3) domain, which interacts with proline-rich motifs present in members of the Wiskott Aldrich syndrome protein (WASP) family and dynamin (26, 42–44). PSTPIP2 lacks the SH3 domain that is indispensable for interaction with WASP compared with PSTPIP1. Instead, it interacts with the C-terminal homology domain of the PEST family phosphatases (45) (**Figure 1**). PSTPIP2 is phosphorylated by tyrosine under the treatment of colony-stimulating factor -1 (CSF-1) (45), and it was also effectively phosphorylated after v-Src transfection (46). Thus, PSTPIP2 could promote the reorganization and chemical stability of the actin cytoskeleton (47, 48).

## The Function of PSTPIP2 in Various Cells

Macrophages and osteoclasts play important roles in AIDs, and the main roles of macrophages and osteoclasts are shown in **Supplement Table 1** (49–52).

### The Function of PSTPIP2 in Macrophages

Macrophages motility are significant in a variety of diseases (53). Their central roles in biological processes are their ability to migrate, reproduce, and devour. Migration, invasion, and phagocytosis are actin-related cellular activities. It has been reported that mutation of PSTPIP2 gene in mice can lead to the occurrence of macrophage-mediated AIDs (16). CSF-1 is the major growth factor that regulates the development and maintenance of macrophages in tissues (53–55). CSF-1 stimulates the reorganization of the actin cytoskeleton and is involved in the chemotactic (56). The CSF-1 receptor tyrosine kinase mediates the effects of CSF-1 (57), which in turn mediates the tyrosine phosphorylation of numerous cellular proteins in response to CSF-1 (58), including that of PSTPIP2 (48).

**Figure 2:**
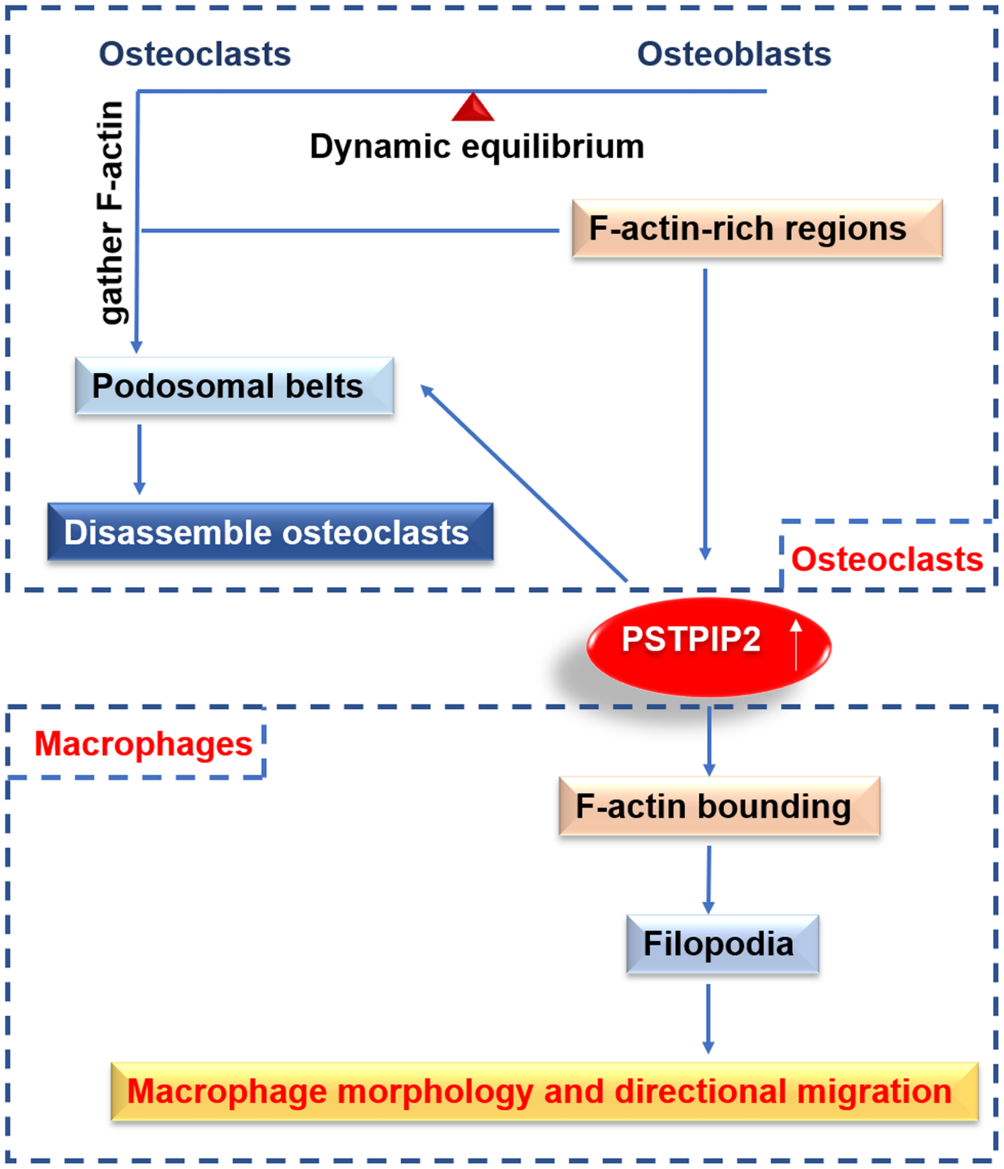
The function of PSTPIP2 in various cells. In macrophages, PSTPIP2 participate in F-actin bundling, promote the formation of filopodia, and then control the morphology and directional migration of macrophages. In osteoclast, PSTPIP2 is highly expressed in F-actin-rich area, and regulate the formation of podosomal belts to promote the decomposition of osteoclast, which plays an important role in the belt dynamic balance of osteoclasts and osteoblasts.

Macrophages are specialized in rapidly changing shape, migration, and phagocytosis. PSTPIP2 is the major tyrosine-phosphorylated protein associated with F-actin in the cytosolic fraction of macrophages stimulated by CSF-1 (45, 48). PSTPIP2 was reported to induce the binding of F-actin and its presence on the actin bundles, indicating that it could interact with newly synthesized actin filaments and package them into bundles *in vitro*. The orientations and chemotaxis of CSF-1 are directly proportional to the expression level of PSTPIP2. Actin bundling is significant for the formation of filopodia (59, 60). Filopodia is thought to affect the direction by acting as environmental sensors (61, 62). PSTPIP2 stimulates an increase in orienteering and chemotaxis, which may partly explain the increased rate of filopodia formation (27). PSTPIP2 is an actin bundling protein that controls the structure of the actin cytoskeleton, and hence controls the morphology and movement of macrophages downstream of CSF-1R (27) (**Figure 2**). The coil region may be significant for the above function of PSTPIP2 because it contains a postulated actin-binding domain and is hypothesized to regulate the oligomerization of PSTPIP2.

### The Function of PSTPIP2 in Osteoclasts

Bone remodeling is a key process in the development, maintenance, and repair of vertebrates. It involves the synergistic activity of osteoblasts and osteoclasts during bone digestion (63). An imbalance of activity between the two cell types can lead to disabling illnesses such as osteopenia or osteoporosis (64).

Osteoclasts are multinucleated cells produced by hematopoietic mononuclear precursors. Bone resorption in vertebrates depends on the ability of osteoclasts to gather F-actin podosomes that coalesce into podosomal belts, forming sealing tapes. Sealing tapes separate bone-facing ruffled membranes from other membranes and disassemble osteoclasts as they migrate to new areas (65). Thus, cycles of cell migration and bone digestion are related to the assembly and disassembly of F-actin-rich structures (66).

It has been reported that PSTPIP2 is highly expressed in the F-actin-rich sealing area, and it can regulate the podosomes to form the sealed area. PSTPIP2 plays important roles in the homeostasis of osteoblasts and osteoclasts (63) (**Figure 2**). PSTPIP2 presents a typical F-BAR domain, which contains proteins that sense the positive membrane curvature and can generate membrane tubules *in vitro* (67–69). This structure can also explain the role of dynamin in podosome dynamics. Dynamin is a Src-dependent GTPase that aggregates on the tubular membrane during clathrin-mediated endocytosis (70). In addition to serving as a membrane scaffold, PSTPIP2 is also used as a docking platform for recruiting podosome components in osteoclasts (63). PSTPIP2 binds to talin1, which binds αvβ3 integrin and F-actin, and is critical for bone degradation (71). PSTPIP2 can also be used as a docking platform for tyrosine-protein phosphatases PTPN12 and PTPN22, contributing to the stabilization of podosomes and sealing areas in osteoclasts.

## Roles of PSTPIP2 in Inflammatory Diseases

### Chronic Recurrent Multifocal Osteomyelitis

Chronic recurrent multifocal osteomyelitis (CRMO) is an inflammatory disease characterized by recurrent fever and bone pain, resulting from inflammation (6) caused by aseptic osteomyelitis (72–74). CRMO patients are mostly children, and the clinical manifestations are bone inflammation, destruction, and deformity (75). Plain films can be normal in the early stages of the disease. However, they usually present as sclerosing osteolytic lesions in the long bone around the growth plate (76, 77).

**Figure 3:**
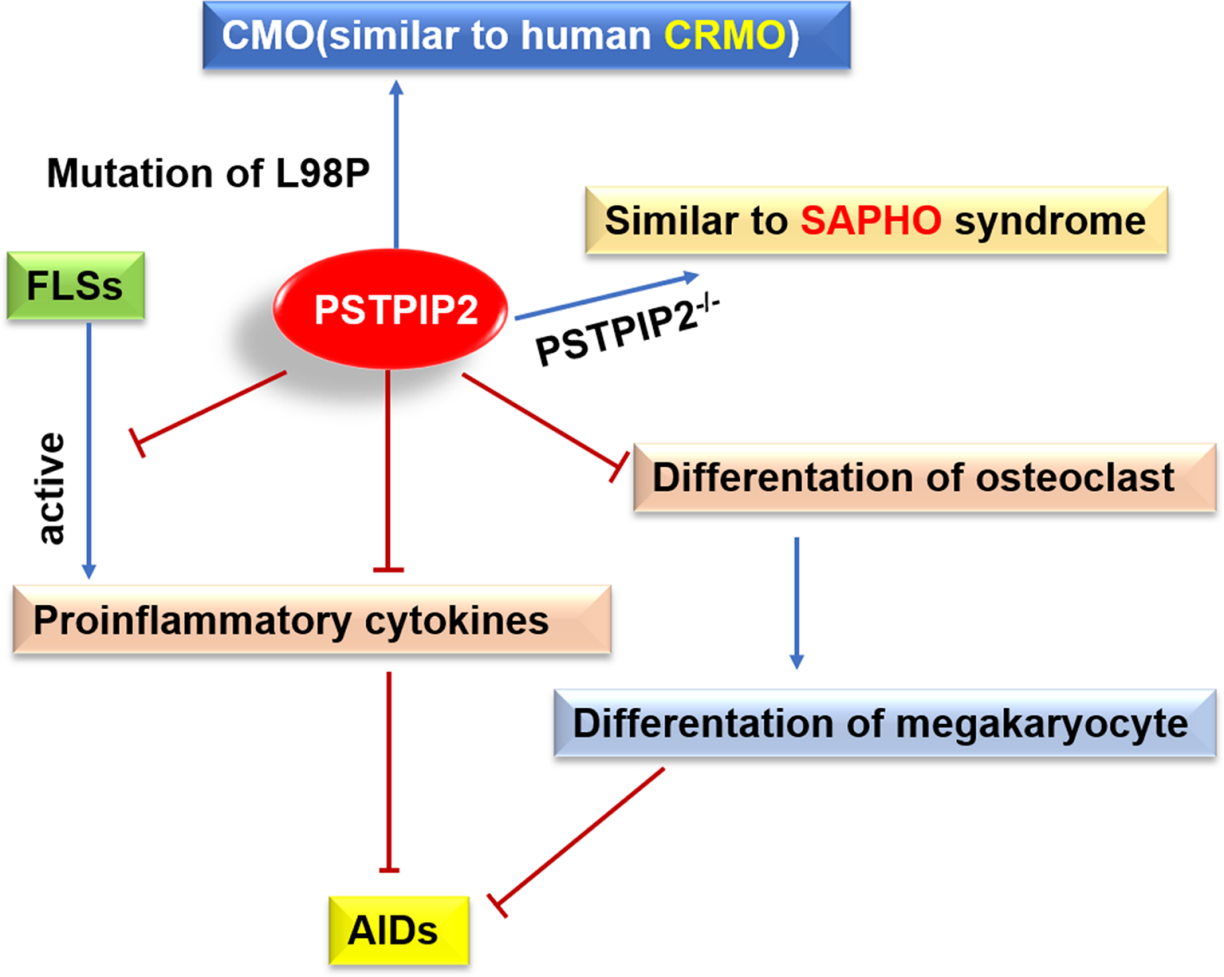
Possible roles of PSTPIP2 in AIDs. Mutation of L98P in PSTPIP2 caused CMO that similar to human CRMO, and PSTPIP2^-/-^ mice exhibit an inflammatory response similar to SAPHO syndrome. PSTPIP2 can inhibit the activation of FLSs, the production of proinflammatory cytokines and the differentiation of osteoclasts, leading to hinder the occurrence of AIDs.

It is widely accepted that inflammatory immune cells play important roles in the induction and maintenance of various inherited and induced bone diseases (78). CRMO is a complex genetic disease and several genes that cause sterile osteomyelitis have been identified in human and animal models, including PSTPIP2. The missense mutations in PSTPIP2 leaded to osteomyelitis and osteopathy with bone deformities in mice (17, 23, 38) (**Figure 3**). As mice cmo disease, which is similar to the experimental model of human CRMO, is specifically mediated by the high production of IL-1β of neutrophils (79), an autoinflammatory osteomyelitis mediated by caspase-8/NLRP3 inflammasome driven by the nonreceptor tyrosine kinase SYK (80). IL-1 is a major mediator of innate immunity and is considered a major cytokine in local and systemic inflammation (81). IL-1 plays a key role in the pathogenesis of AIDs. The inhibition of IL-1 has become a key therapeutic target. In addition, it has been reported that PSTPIP2 can also negatively regulate the ROS generation pathway of neutrophil NOX2 NADPH oxidase. NADPH oxidase dysregulation promotes bone damage of autoinflammatory osteomyelitis (82).

### Synovitis, Acne, Pustulosis, Hyperostosis, and Osteitis

SAPHO syndrome is a relatively obscure autoinflammatory condition (83). Most patients with SAPHO syndrome are children and young adults, particularly females (84). The patient with SAPHO presents with significant bone and joint involvement (85, 86). The skin lesion of SAPHO syndrome is a neutrophilic dermatosis (87). SAPHO syndrome has been associated with CRMO and multiple aseptic inflammatory bone lesions (88). Although the pathogenesis remain unclear, SAPHO syndrome tends to be familial and is associated with genetic abnormalities, and there is an increasing understanding that SAPHO is similar to other AIDs (38, 89). The discovery of familial clustering cases supported the genetic basis of SAPHO, but no specific mutations were found in SAPHO patients (90).

PSTPIP2 participates in macrophage activation, neutrophil migration, cytokine production, and osteoclast differentiation (91). It has been hypothesized to play crucial roles in innate immune diseases and autoinflammatory bone disorders, including SAPHO. PSTPIP2 knockout mice (PSTPIP2^-/-^) have been reported to develop paw swelling, synovitis, osteoproliferation, and osteitis, similar to SAPHO syndrome (91) (**Figure 3**). Studies have shown that variants rs10889677, rs2201841, and rs7517847 of IL-23R, and variant rs2243248 of IL-4, showed strong associations with SAPHO syndrome. Patients carrying the A-G-C-G-T haplotype of IL-23 are significantly more likely to develop SAPHO syndrome (92).

## Potential Involvement of PSTPIP2 in Inflammatory Diseases

**Figure 4:**
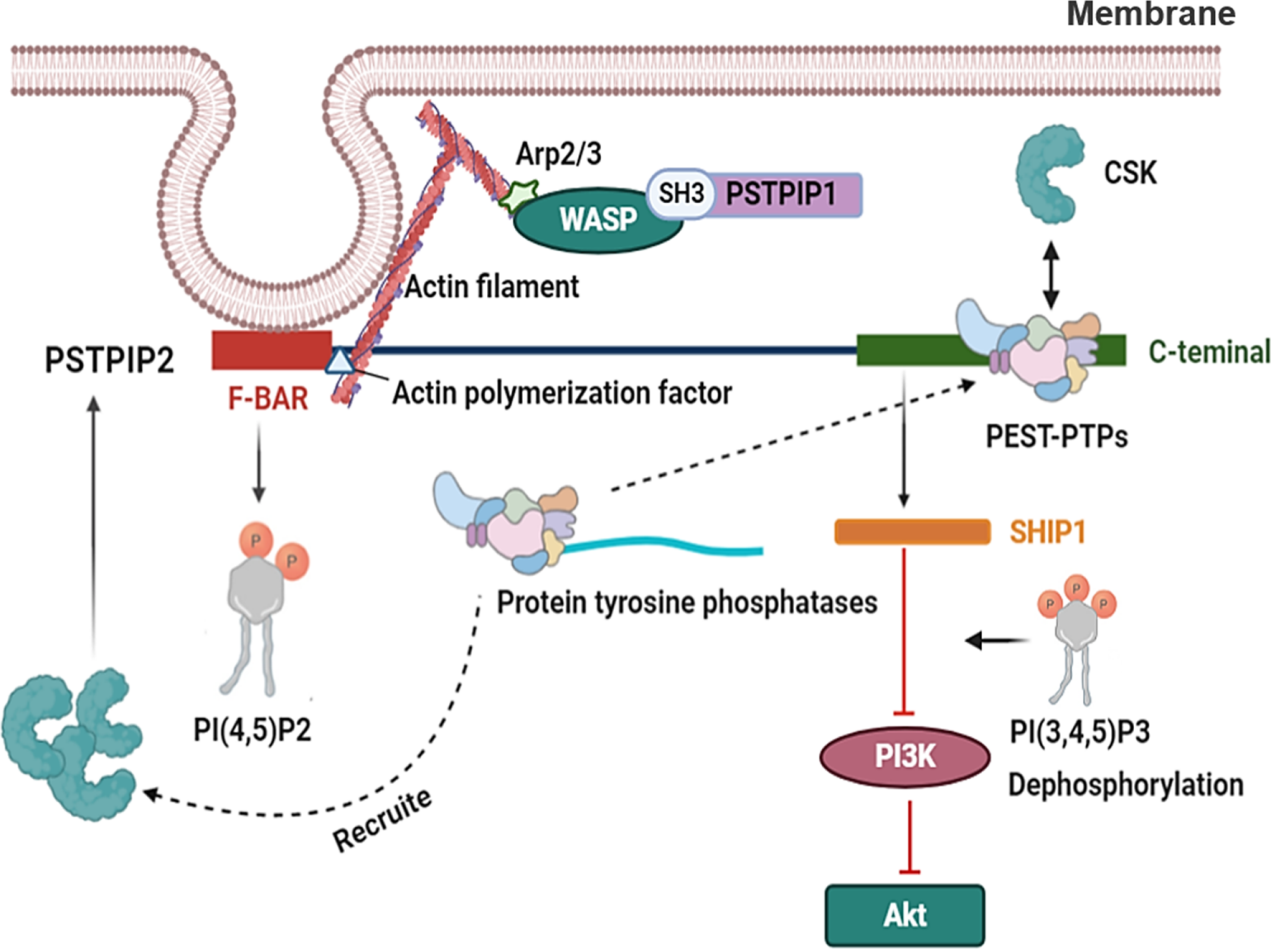
The related mechanism of PSTPIP2. PSTPIP2 have a N-terminal F-BAR domain and a C-terminal tail. The PSTPIP2 C-terminal can be combined with PEST- family and the inhibition of PSTPIP2 is mediated by protein tyrosine phosphatases located in the central area of PEST-family. PEST-PTPs can bind to CSK through a proline-rich or tyrosine-rich motif in their central region. PEST-PTPs also involves recruiting CSK to PSTPIP2, which further enhances the independent binding of CSK to PSTPIP2. SHIP1 binds to the key tyrosine residues at the C terminal of PSTPIP2. Lipid phosphatase SHIP1, a regulatory protein, controls the activity of the PI3K pathway *via* dephosphorylating its crucial mediator, phosphatidylinositol-3,4,5-trisphosphate [PI (3,4,5) P3]. This results in the decreased activity of some of its downstream effectors, such as Akt. PSTPIP2 regulate membrane curvature by binding to cell membrane phospholipids by F-BAR domain. F-BAR could bind to actin polymerization factor of actin filament. WASP binds to the Arp2/3 complex and triggers the formation of a weave of actin comprising branched actin filaments. And the SH3 domain of PSTPIP1 could interact with WASP.

PSTPIP2 is known to play an important role in the development of AIDs, where its reduced or complete loss of expression is the major cause of the diseases (16, 17, 38). However, the mechanisms controlling the function of PSTPIP2 have not been fully elucidated. As an adaptor protein, it may function by regulating interactions between proteins, inhibitory enzymes, and other negative regulators (**Figure 4**). Partial inhibition of PSTPIP2 is mediated by protein tyrosine phosphatases in the PTP-PEST family. These tyrosine phosphatases interact with the central part of the protein. The only known PSTPIP2 binding molecule in this class is PEST-PTPs (23, 46), which consists of three members: PEP/LYP, PTP-HSCF (PTPN18), and PTP-PEST (PTPN12) (93). All three family members can bind to CSK through a proline-rich or tyrosine-rich motif in their central region (94–96).

Another possible role of PEST-PTPs is the recruitment of CSK to PSTPIP2, which further enhances the independent binding of CSK to PSTPIP2. In addition, more evidence supports the importance of PEST-PTPs in PSTPIP2 functionality. These adaptor proteins are involved in the regulation of inflammation, and changes in their function lead to the development of autoinflammatory diseases (17, 37). For example, mutation of W232A in PSTPIP2 prevents its binding to PEST-family phosphatase, and impairs the ability of PSTPIP2 to restrain the differentiation of osteoclasts and megakaryocytes (23, 97). However, other areas of PSTPIP2 that are not required for PEST-family phosphatase binding have also been shown to be indispensable for PSTPIP2 function. It has been reported that PSTPIP2 can bind to the inhibitory enzymes CSK and SHIP1 (98).

SHIP1 binds to the key tyrosine residues at the C terminal of PSTPIP2, which is important for the independent inhibition of PEST-phosphatase in different cellular systems. This region is important for PSTPIP2-mediated inhibition of IL-1β processing in neutrophils, and SHIP1 inhibition led to an enhancement of this process. Lipid phosphatase SHIP1, a regulatory protein, controls the activity of the PI3K pathway by dephosphorylating its crucial mediator, phosphatidylinositol-3,4,5-trisphosphate [PI (3,4,5) P3]. This results in decreased activity of some of its downstream effectors (99). A typical example of this effect is Akt, a serine/threonine kinase, which is involved in cell activation, proliferation, metabolism, and survival, and is absorbed into the plasma membrane by PI(3,4,5)P3 for further activation (100). There are also ample data to show that SHIP1 negatively regulates the MAPK pathway through various mechanisms (101).

The protein tyrosine kinase CSK is another key negative regulator of leukocyte signaling. PSTPIP2 is characterized by an N-terminal F-BAR domain that mediates interactions with membrane phospholipids as well as a C-terminal tail containing various interaction motifs (102). The F-BAR domain of PSTPIP2 can interact with PI (4,5) P2 (24), and its C-terminal could bind PEST-PTPs through an interactive motif including tryptophan 232 (23, 103). The C-terminus of PSTPIP2 contains several unknown functional tyrosine, which are often phosphorylated in macrophages after exposure to M-CSF (45).

PSTPIP2 deficiency results in an increased response to CSF-1 stimuli, and causes the overactivation of Erk1/2 and STAT1 in macrophages (16); Erk1/2 is the main regulator of proliferation of macrophages and their progenitors induced by CSF-1 (104). Therefore, PSTPIP2 suppresses inflammation and osteoclast generation as a CSF-1R signaling negative feedback regulator. Overexpression of PSTPIP2 restrains membrane ruffling and formation of filopodia, cell stretching, and motility (27). PSTPIP2 might play a vital role in the development of AIDs, and knowledge of these mechanisms will be useful for therapies for AIDs. In summary, PSTPIP2 may be a potential therapeutic target for AIDS.

## Conclusions

Proline-serine-threonine-phosphatase-interacting protein 2 (PSTPIP2) belongs to the F-BAR family of proteins. There is evidence that PSTPIP2 plays an important role in the development of AIDs. The PSTPIP2^cmo^ mouse strain is a character model for this type of disease. Due to a mutation that resulted in a deficiency of the adaptor protein PSTPIP2, these animals developed chronic inflammatory multifocal osteomyelitis, with symptoms are similar to human CRMO. In macrophages, PSTPIP2 can negatively regulate the activation of macrophages and inhibit the release of inflammatory cytokines, thereby inhibiting the development of inflammation. In osteoclasts, PSTPIP2 plays an important role in the homeostasis of osteoblasts and osteoclasts and participates in osteoclast-induced osteoclasts. This plays a significant role in the occurrence and development of AIDs, such as CRMO and SAPHO. In addition to macrophages, neutrophils, monocytes, natural killer (NK) cells, T cells and B cells also play important roles in autoinflammatory diseases (4, 105), and the study of PSTPIP2 in these cells remains to be explored. The mechanisms controlling the function of PSTPIP2 have not been fully elucidated.

Partial inhibition of PSTPIP2 is mediated by protein tyrosine phosphatases in the PEST-family, and more evidence supports the importance of PEST-PTPs to PSTPIP2 functionality. It has been reported that PSTPIP2 can bind to the inhibitory enzymes CSK and SHIP1. Another possible role of PEST-PTPs is the recruitment of CSK to PSTPIP2, which further enhances the independent binding of CSK to PSTPIP2. These adaptor proteins are involved in the regulation of inflammation, and changes in their function lead to the development of autoinflammatory diseases. PSTPIP2 also plays an important role in liver fibrosis (18). Overexpression of PSTPIP2 inhibited the expression of M1 markers by suppressing STAT1 activity and enhanced the expression of M2 markers by promoting STAT6 activity (**Supplement Table 2**). AIDs are spontaneous inflammatory diseases caused by innate immune system disorders. Accumulating evidence suggests that PSTPIP2 of the F-BAR family plays a vital role in AIDs, they may be involved in controlling inflammation and the pathophysiology of some autoinflammatory syndromes.

Actin is a major component of the cellular dielectric structure, and abnormal regulation of the cytoskeleton determines complex clinical manifestations, inflammatory manifestations, and immune deficiency. “Actinopathies”, a new area of AIDs, is a significant overlap between autoinflammation and immune-dysregulation. Autoinflammatory periodic fever, immune deficiency, and thrombocytopenia (PFIT) syndrome are caused by mutations in the actin-regulating *WDR1* gene that encodes WD-repeat 1 (106). The protein plays a critical role in the dynamic reorganization of the cytoskeleton. A new monogenic AIDs characterized by IL-18 hypersecretion have been associated with cytoskeletal abnormalities (107). PSTPIP2 exhibits F-actin bundling activity, indicating that it plays a certain role in the membrane–cytoskeleton interface.

There is growing evidence that mutations in different genes associated with autoinflammation are associated with other “neutrophilic diseases” (108–110). Pyoderma gangrene (PG) is a rare inflammatory neutrophilic skin disease that may occur in the context of PAPA (pyogenic sterile arthritis with PG and acne) and SAPHO syndromes as well as PASH (pyoderma gangrenosum, acne and pyogenic hidradenitis) syndromes (108). Two mutations encoding PSTPIP1 gene (A230T and E250Q) were found in PAPA patients (111), and the absence of PSTPIP2 triggers SAPHO syndromes (91). Neutrophil-mediated dermatosis are now considered to be a spectrum of polygenic autoinflammation (112).

From the above, PSTPIP2 is mainly expressed in macrophages and regulates the functions of macrophages and osteoclasts, affecting the occurrence and development of AIDs. PSTPIP2 may be a potential target for the treatment of AIDs, providing a new theoretical support for the treatment of AIDs. And it is speculated that PSTPIP2 may play an important role in actinopathies. Autoinflammatory diseases and autoimmune diseases share many similar etiological and clinical features, including genetic susceptibility and recurrent systemic inflammatory flares (113). It has been reported that PSTPIP2 suppressed inflammation in autoimmune diseases (114). The previous research of our group indicated that adding PSTPIP2 expression alleviated liver fibrosis and hepatic inflammation in mice (18). PSTPIP2 may be closely related to autoimmune diseases and inflammation-related diseases. More information is clearly needed to fully understand the functions, mechanisms, and regulation of PSTPIP2 under normal and pathological conditions. However, there are few studies on the role of PSTPIP2 in AIDs. Therefore, additional research addressing these issues is warranted to better understand PSTPIP2 and to fully recognize the similarity and specificity of members of the PSTPIP family.

## Author Contributions

J-JX and H-DL writing, original draft and figure preparation. X-SD and J-JL original draft preparation. X-MM and CH table preparation and editing. JL writing, review, and editing. All authors contributed to the article and approved the submitted version.

## Funding

This work was supported by grants from the National Natural Science Foundation of China (No. 81770609), Anhui Medical University of Science and Technology (No. 1704a0802161), and The University Synergy Innovation Program of Anhui Province (No. GXXT-2019-045).

## Conflict of Interest

The authors declare that the research was conducted in the absence of any commercial or financial relationships that could be construed as a potential conflict of interest.

## Abbreviations

AIDs, Autoinflammatory diseases; CIP4, CDC42-interacting protein 4; cmo, chronic multifocal osteomyelitis; CRMO, Chronic recurrent multifocal osteomyelitis; CSF-1, Colony-stimulating factor-1; CTH, Carboxy-terminal homology; F-BAR, Fes/CIP4 homology-Bin/Amphiphysin/Rvs; FLSs, Fibroblast-like synoviocytes; FMF, Familial mediterranean fever; PAPA, Pyogenic Arthritis, Pyoderma gangrenosum, Acne; PEST- family, Proline-, glutamic acid-, serine- and threonine-rich family; [PI(3,4,5)P3], Phosphatidylinositol-3,4,5-trisphosphate; PSTPIP, Proline-serine-threonine-phosphatase-interacting protein; SAPHO, Synovitis, acne, pustulosis, hyperostosis and osteitis; SH3, Src homology 3; WASP, Wiskott Aldrich syndrome protein.
