# Supplementary figures and images for: Role of the F-BAR Family Member PSTPIP2 in Autoinflammatory Diseases
Source: Front Immunol. 2021 Jun 28;12:585412. doi: 10.3389/fimmu.2021.585412 (PMC8273435; doi:10.3389/fimmu.2021.585412)

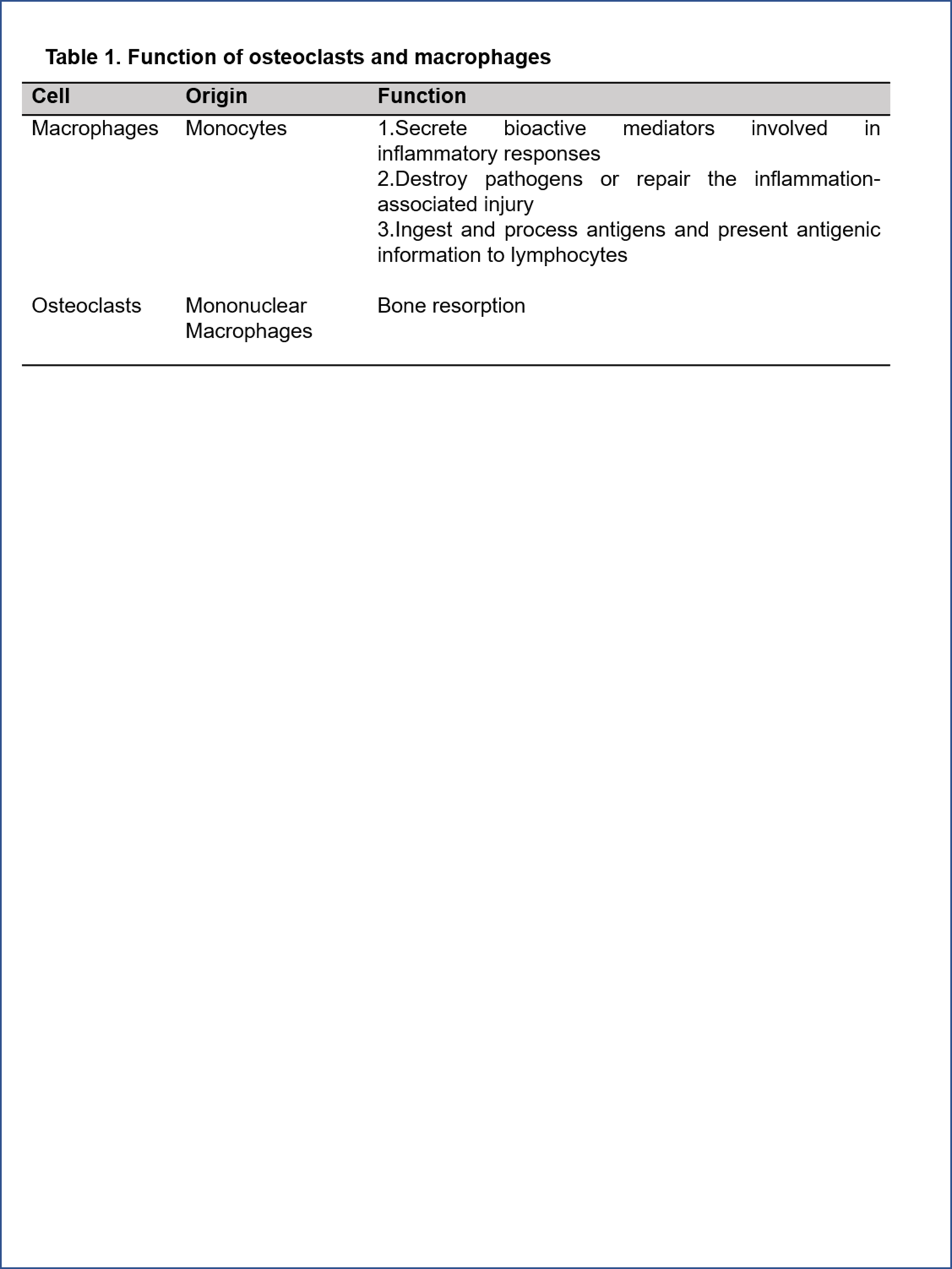

Supplement: Supplementary file 1 [file Image_1.tif]

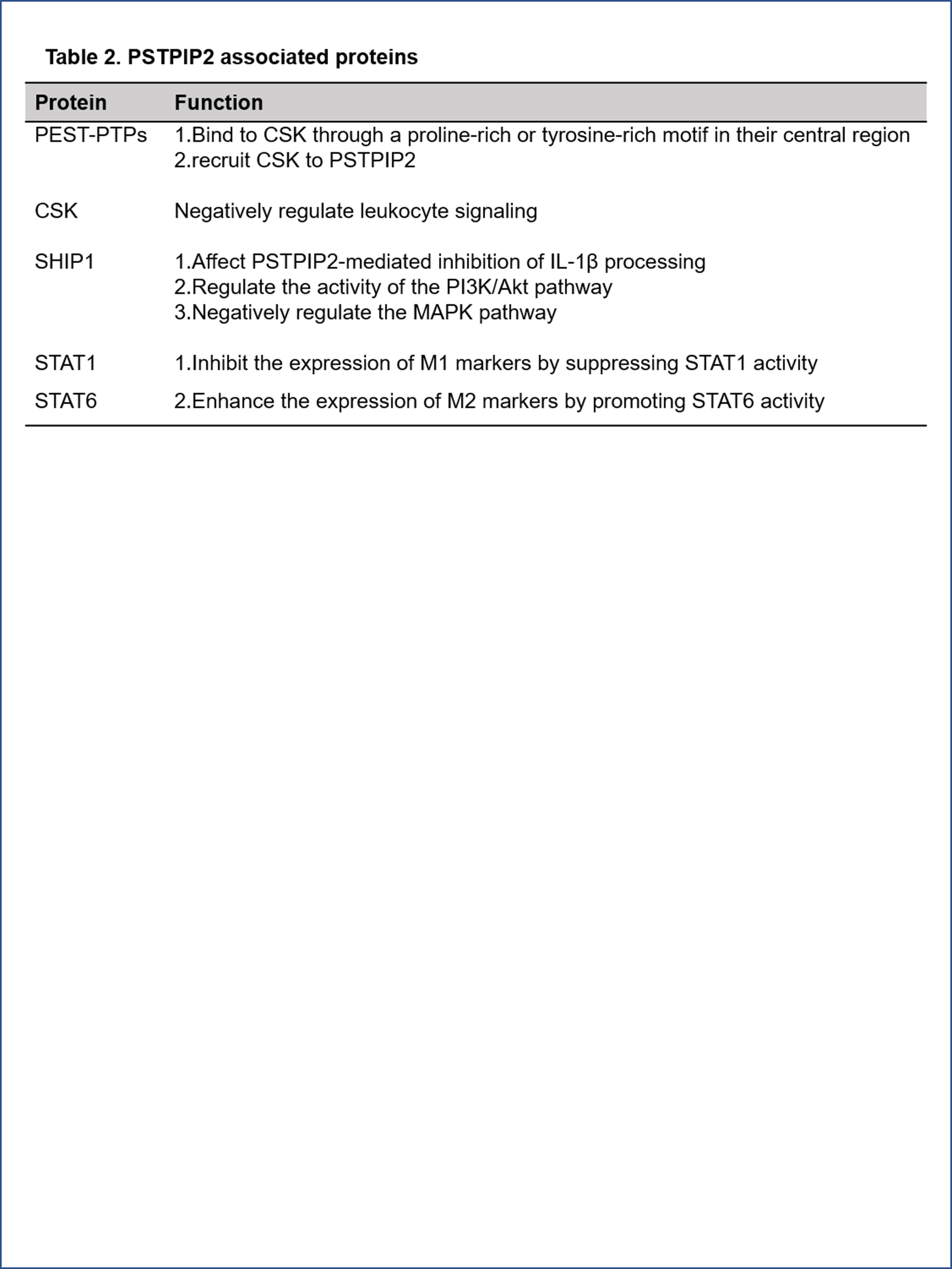

Supplement: Supplementary file 2 [file Image_2.tif]
